# Vitamin and mineral supplements in pregnancy and the risk of childhood acute lymphoblastic leukaemia: a case-control study

**DOI:** 10.1186/1471-2458-7-136

**Published:** 2007-07-03

**Authors:** John D Dockerty, Peter Herbison, David CG Skegg, Mark Elwood

**Affiliations:** 1 Department of Preventive and Social Medicine, University of Otago, PO Box 913, Dunedin, New Zealand; 2 BC Cancer Research Centre, Cancer Control Research, 675 West 10^th ^Ave, Vancouver, BC, V5Z 1L3, Canada

## Abstract

**Background:**

An earlier case-control study from Western Australia reported a protective effect of maternal folic acid supplementation during pregnancy on the risk of childhood acute lymphoblastic leukaemia (ALL). The present study tested that association.

**Methods:**

A national case-control study was conducted in New Zealand. The mothers of 97 children with ALL and of 303 controls were asked about vitamin and mineral supplements taken during pregnancy.

**Results:**

There was no association between reported folate intake during pregnancy and childhood ALL (adjusted odds ratio (OR) 1.1, 95% confidence interval (CI) 0.5–2.7). Combining our results with the study from Western Australia and another study from Québec in a meta-analysis gave a summary OR of 0.9 (95% CI 0.8–1.1).

**Conclusion:**

Our own study, of similar size to the Australian study, does not support the hypothesis of a protective effect of folate on childhood ALL. Neither do the findings of the meta-analysis.

## Background

Despite decades of research, the aetiology of childhood leukaemia remains enigmatic. There have been clear increases in the incidence rates of childhood leukaemia in New Zealand and other countries, highlighting the aetiological importance of unknown environmental factors [1]. There is a natural appeal to any evidence suggesting that a modifiable factor could be protective. In 2001, a case-control study from Western Australia showed a protective effect of maternal folate supplements (taken in pregnancy) on the risk of childhood common acute lymphoblastic leukaemia (ALL) [2]. The odds ratio (OR) for iron or folate supplementation was 0.4 (95% confidence interval (CI) 0.2–0.7). Most mothers used folate in conjunction with iron, so the authors could not report separate findings for folate alone. They suggested that the effect was likely to be from the folate or from iron and folate together.

A case-control study from northern California looked at maternal diet and vitamin supplements in the twelve months before pregnancy, and found no statistically significant association between childhood ALL and any vitamin or iron supplements [3]. Total dietary folic acid, presumably including supplements, gave an odds ratio of 0.8 (95% CI 0.3–1.8). However use of folic acid supplements specifically was not reported, so the Californian findings are not directly comparable with those from Australia. In a large case-control study of childhood ALL from Québec, Shaw and colleagues looked at maternal use of vitamins and minerals during pregnancy [4]. They found no association between use of supplements that contained folic acid (alone or in combination) and childhood ALL in the offspring (OR 1.0, 95% CI 0.8–1.2).

In this paper we have tested the hypothesis raised by the Australian study that folate supplementation in pregnancy reduces the risk of childhood ALL. We have also assessed the effects of iron and multivitamin supplements. In addition to looking at maternal pregnancy consumption, we have looked at supplement use by the child.

## Methods

In the 1990s, we interviewed families for a case-control study of childhood cancers in New Zealand to test hypotheses related to infections and vaccinations, electromagnetic fields, chemicals and other exposures [5,6]. The study included questions for mothers about the use of vitamins and other supplements.

The detailed methods of this population-based national case-control study are described elsewhere [5,6]. Briefly, the cases were ascertained from the National Cancer Register and other sources. The whole study included children diagnosed with any type of cancer at ages 0–14 during 1990–93. The 344 eligible cases were born and resident in NZ. They had been diagnosed with cancer of any kind; including 131 children with leukaemias and 213 with solid cancers. The mothers of 303 cases (88 per cent) gave interviews. Restricting to ALL, there were 104 eligible cases and 97 mothers (93 per cent) gave interviews.

The controls were selected at random from birth records, while matching 1:1 to cases on age and sex. Of 303 eligible first choice controls, the mothers of 209 (69%) consented and took part. Replacement controls were selected for the rest [5,6].

Home interviews were conducted using structured questionnaires. Mothers were asked "*Did you take any vitamins or mineral supplements during your pregnancy, in the 3 months before, or while breastfeeding? Include iron or folate and any others*." Those who replied 'yes' were then asked "*What was the name of the vitamin or mineral (please be specific)?*" They were then asked to specify their usage of each vitamin/mineral in each of the periods of interest. Information was also collected on vitamin and mineral supplementation of the child. Mothers were asked: *"Did {child's name} take any vitamins or mineral supplements for five days or more (or on 5 or more occasions) at any time prior to ...../...../..... {reference date}?" *For each case, the reference date was their diagnosis date. For each control it was the date on which they were the same age (in days) as their matched case was at diagnosis. If the mother said 'yes' to the question about supplement use by the child, then she was asked to specify the name of each vitamin or mineral supplement taken, and to answer questions about the timing, duration and frequency of its use. We excluded supplements taken by the child within 6 months of the diagnosis/reference date because they may have been taken as a consequence of early disease and were not likely to be related to causation.

Folate could be taken on its own, or more usually in combination with iron or as part of a multivitamin preparation. Separate analyses were attempted for 'any folate' (incorporating all of the aforementioned), and for 'folate only' if numbers permitted.

The main analysis involved the cases of ALL and controls, and was by unconditional logistic regression. This unmatched analysis was based on the cases and all the available controls, to increase the statistical power (97 ALL cases, 303 controls). In breaking the matching, we always adjusted for the matching factors (age and sex). Possible confounders were identified on the basis of plausibility and a 10% change in estimate. Such a strategy was used for other aspects of the study [5,6]. Following this strategy, the mother's marital status and education were adjusted for in addition to the matching factors. Matched analyses were also conducted to check whether they would have made any difference to the results.

## Results

Folic acid supplements were taken in pregnancy by 9% of case mothers and 9% of control mothers. Among the controls, only one of the 27 women who took folate (Table 1) did not take iron, whereas 116 of the 142 who took iron did not take folate. Among the cases, two of the eight women who took folate did not take iron, and 38 of the 44 who took iron (Table 1) did not take folate. The correlation coefficient between iron and folate use in the mothers was 0.27, and in the children it was 0.93. There was no indication from either the matched or unmatched analyses of an interaction between iron and folate supplementation. Only 3 case mothers and 14 control mothers took multivitamins. All of those were recorded as having taken folate and iron. All of the multivitamins that were taken included folate and iron. Among the first choice controls, 9.8% of mothers took folate supplements, whereas in the second or later choice controls this was 7.7%; the difference was not significant (p = 0.56).

**Table 1:** Acute lymphoblastic leukaemia in relation to intake of vitamin and mineral supplements by the mother during pregnancy, and by the child before the reference date. Unmatched analyses

**Supplement & details of use**	**Categories**	**No. of cases**	**No. of controls**	**Odds ratio (CI)**	
				
				***Adjusted for age in years and sex only***	Adjusted for age, sex, and other variables *
***Mother's use during the pregnancy***					
Folic acid (any,	No	82	268		
with or without iron)	Yes	8	27	0.9 (0.4–2.2)	1.1 (0.5–2.7)
Iron (any,	No	45	151		
with or without folic acid)	Yes	44	142	1.1 (0.7–1.8)	1.2 (0.7–2.1)
Iron without folic acid	No	51	177		
	Yes	38	116	1.2 (0.7–2.1)	1.3 (0.8–2.3)
Multivitamins	No	87	281		
	Yes	3	14	0.7 (0.2–2.5)	0.8 (0.2–3.1)
Other vitamin or	No	78	263		
mineral supplements	Yes	13	32	1.3 (0.6–2.6)	1.5 (0.7–3.1)

***Child's use before the reference date ***†					

Folic acid (any,	No	90	288		
with or without iron)	Yes	6	15	1.2 (0.4–3.2)	1.0 (0.4–2.8)
Iron (any,	No	89	286		
with or without folic acid)	Yes	7	17	1.2 (0.5–3.1)	1.1 (0.4–2.8)
Iron without folic acid	No	95	301		
	Yes	1	2	1.3 (0.1–15.5)	1.6 (0.1–19.3)
Multivitamins	No	90	288		
	Yes	6	15	1.2 (0.4–3.2)	1.0 (0.4–2.8)
Other vitamin or	No	82	272		
mineral supplements	Yes	14	31	1.5 (0.7–3.0)	1.6 (0.8–3.4)

* Adjusted for age, sex, marital status, and mother's education.

† Restricted to child's use of a supplement for 5 or more days, either in a row or separate days. Child's usage in the six months prior to the diagnosis or reference date was not counted.

The unmatched analyses showed no statistically significant association between the mother's use of folate (any, with or without iron) in pregnancy and the risk of childhood acute lymphoblastic leukaemia (Table 1, OR 1.1, 95% CI 0.5–2.7). There were also no associations relating to intake during the other periods studied (3 months pre-pregnancy and while breastfeeding). There are small numbers in some categories and the estimates are imprecise. Folate 'only' (without iron) could not be examined because of the low prevalence of this exposure; this was also the case in the study by Thompson and colleagues. When we restricted the diagnosis to B-precursor ALL, there was some loss of precision, but no material change to the findings. Matched analyses also showed no statistically significant relationships, but they produced wider confidence intervals due to a loss of power (conditional logistic regression, data not shown).

There were no statistically significant findings relating to the mother's use of iron (without folate) or multivitamins (Table 1). The odds ratios for iron were above 1.0, and those for multivitamins were below 1.0. When the regressions for folate were adjusted for iron and vice versa the results did not change. No associations were found between the child's use of folate, iron or multivitamins and the risk of ALL (Table 1).

## Discussion

Since the report by Thompson and colleagues [2], several studies have investigated the risk of paediatric ALL in relation to variant forms of the methylenetetrahydrofolate reductase (MTHFR) gene. Not all these studies have been consistent. Some have suggested a decreased risk of ALL for some patients with particular allelic variants [7], while others have not [8,9].

The authors of the case-control study from California reported no association between maternal pre-pregnancy dietary folate and childhood ALL [3]. They also found no association for iron. A subsequent paper from that study, with an expanded number of cases and controls, reported a decreased risk of ALL in relation to use of iron supplements by mothers in the period 3 months before pregnancy, during pregnancy or while breastfeeding, with an overall odds ratio of 0.7 (0.5–0.9) [10]. Although we found no associations with iron use (Table 1), several other studies have reported varying findings [10].

Shaw et al. have reported findings from their large Canadian case-control study of childhood ALL and use of medications during pregnancy [4]. They included information about vitamins – those containing folic acid (alone or in preparations containing other vitamins or minerals) and other vitamins or minerals. Almost 60 percent of the mothers in their study used pregnancy supplements that contained folic acid. They found no association between the risk of ALL and maternal use of supplements containing folic acid (OR 1.0, 95% CI 0.8–1.2), or other vitamins (OR 1.0, 95% CI 0.7–1.3) [4].

Limitations of our study include its small size (a consequence of the small population of New Zealand), and the low prevalence of folate supplementation reported by the mothers of the cases and controls. On its own, our study cannot exclude a protective association like that indicated by the Australian study – this is shown quantitatively by the overlap in the confidence intervals of the two studies. As in the Australian study, it was not possible to look at the effect of folate alone (without iron). We also lacked information on dietary folate. Fortification of foods such as breakfast cereals began in the mid 1990s, after the data collection for our study [11]. Folate supplement use in the periconceptional period was to some extent promoted following the publication of the results of the MRC trial on folic acid and prevention of neural tube defects in 1991. However New Zealand did not have a comprehensive folic acid campaign like that in the UK [12].

Conducting a fixed effects meta-analysis [13] to combine the Thompson and Shaw studies with our study, the joint folate analysis gives an OR of 0.9 (95% CI 0.8–1.1). In other words, there is no association between the risk of childhood ALL and maternal folate use in pregnancy for the three studies combined. The heterogeneity test gives I^2 ^= 74% (p = 0.02) because the findings of the Thompson study differ from the other two.

Several possible biological mechanisms have been suggested for an effect of folate in reducing the risk of cancers. These relate to alterations in DNA methylation, a role of folate in DNA repair, methylenetetrahydrofolate reductase polymorphisms, and other possible mechanisms [14]. Our results did not confirm a protective effect of folate supplementation in pregnancy on the risk of childhood ALL. Retrospective cohort or nested case-control studies could be considered in countries where it is possible to link cancer registrations with antenatal clinic records. But these would be limited by the quality of data recorded about supplement prescribing or usage, and they could not easily take account of 'over-the-counter' purchasing of supplements by mothers.

## Conclusion

We did not confirm the association found in the Australian study of a lower risk of ALL related to maternal folic acid supplementation in pregnancy. Our meta-analysis of three relevant studies showed no statistically significant association.

## Abbreviations

ALL, acute lymphoblastic leukaemia. OR, odds ratio. CI, confidence interval

## Competing interests

The author(s) declare that they have no competing interests.

## Authors' contributions

JD, ME and DS designed the case-control study. JD co-ordinated the data collection; and checked and cleaned the data with assistance from PH. PH and JD conducted the analyses, which were interpreted by all the authors. JD wrote the report, and all the authors contributed to revisions.

## Pre-publication history

The pre-publication history for this paper can be accessed here:


